# Elucidating the factors and consequences of the severity of rumen acidosis in first-lactation Holstein cows during transition and early lactation

**DOI:** 10.1093/jas/skae041

**Published:** 2024-02-14

**Authors:** Thomas Hartinger, Ezequias Castillo-Lopez, Nicole Reisinger, Qendrim Zebeli

**Affiliations:** Institute of Animal Nutrition and Functional Plant Compounds, University of Veterinary Medicine, 1210 Vienna, Austria; Christian Doppler Laboratory for Innovative Gut Health Concepts of Livestock, 1210 Vienna, Austria; Institute of Animal Nutrition and Functional Plant Compounds, University of Veterinary Medicine, 1210 Vienna, Austria; Christian Doppler Laboratory for Innovative Gut Health Concepts of Livestock, 1210 Vienna, Austria; dsm-firmenich, AHN R&D Center Tulln, 3430 Tulln, Austria; Institute of Animal Nutrition and Functional Plant Compounds, University of Veterinary Medicine, 1210 Vienna, Austria; Christian Doppler Laboratory for Innovative Gut Health Concepts of Livestock, 1210 Vienna, Austria

**Keywords:** concentrate feeding, cow health, rumen acidosis, rumen fermentation, transition feeding

## Abstract

First-lactation cows are particularly prone to subacute ruminal acidosis (SARA) during transition. Besides common risk factors of SARA, such as feeding of starch-rich diets, an individual severity of SARA in cows has been recently evidenced. Yet, the factors that play a role in SARA severity have not been elucidated. The main goal of this research was to evaluate the factors of SARA severity in first-lactation cows during transition and early lactation, which go beyond high-grain feeding, and to explore their impact on behavior, health, and fermentation in the rumen and hindgut. Twenty-four first-lactation Holstein cows with the same feeding regime were used starting from 3 wk before the expected calving day until 10 wk postpartum. Cows received a close-up diet (32% concentrate) until calving and were then transitioned to a lactation diet (60% concentrate) within 1 week. The SARA severity was assessed by cluster analysis of several rumen pH metrics, which revealed exceptionally longer and more severe SARA in cows denominated as high (*n* = 9), as compared to moderate (*n* = 9) and low (*n* = 6) SARA severity cows (*P *< 0.01). The logistic analysis showed that the length of close-up feeding, age at parturition, and the level of dry matter intake (DMI) were the main factors that influenced the cows’ odds for high SARA severity (each *P *≤ 0.01). Moreover, the ANOVA hinted differences in the metabolic activity of the ruminal microbiome to promote SARA severity, as indicated by highest ruminal propionate proportions (*P* = 0.05) in high SARA severity cows, also with similar DMI. The distinct SARA severity was marginally reflected in behavior and there were no effects of SARA severity or high-grain feeding on blood inflammation markers, which peaked at parturition regardless of SARA severity (*P* < 0.01). Still, ongoing high-grain feeding increased liver enzyme concentrations from 6 wk postpartum on, compared to weeks before (*P *< 0.01), yet irrespectively of SARA severity. In conclusion, first-lactation cows differed in SARA severity under the same feeding regime, which was ascribed to management factors and differences in ruminal fermentation. Further research is warranted to validate these findings and to understand the mechanisms behind differences in the metabolic function of rumen microbiome, in particular in terms of evaluating markers for various SARA severity, as well as to evaluate potential long-term effects on health, performance, fertility, and longevity of dairy cows.

## Teaser

This study revealed that first-lactation dairy cows differ in their susceptibility to subacute rumen acidosis under the same feeding regimen. The susceptibility was related to management factors and not reflected in animal behavior.

## Introduction

Nutrition programs of high-yielding dairy heifers and cows are commonly based on starch-rich diets. Yet, this happens at the expense of physically effective fiber needed to maintain rumen health, and the feeding of such diets is known to trigger digestive disorders including subacute rumen acidosis (SARA; Plaizier et al., 2018). The SARA is characterized as an intermittent drop of ruminal pH below thresholds of 5.6 for more than 180 min/d (Plaizier et al., 2008) or below 5.8 for more than 330 min/d (Zebeli et al., 2008), thus in both cases accounting for the length of the ruminal pH below a certain critical threshold. Penner et al. (2007) used the magnitude of the pH drop to characterize the severity of rumen acidosis, such as ruminal pH between 5.5 and 5.8 indicating mild SARA, between 5.2 and 5.5 indicating moderate SARA, and ruminal pH < 5.2 indicating acute rumen acidosis. Research has suggested that SARA increases the odds of systemic inflammation, laminitis, abomasal displacement, perturbed liver function, and milk fat depression (Humer et al., 2018; Plaizier et al., 2018; Kofler et al., 2023), and this makes SARA a serious metabolic disorder in dairy cattle. It has also been shown that the risk of SARA is especially high during the first weeks after parturition, when cows are abruptly fed high-grain diets and their rumen is not yet adapted, coupled with the stressors of endocrine and metabolic changes, milking, rehousing, or potential postpartum complications (Penner et al., 2007; Vergara et al., 2014; Zebeli et al., 2015).

Recent data have further demonstrated that parity constitutes a central risk factor for SARA, whereby first-lactation cows are particularly vulnerable to experience more severe SARA episodes and eventually suffer from associated health implications (Stauder et al., 2020; Khorrami et al., 2021). This may be related to the fact that first-lactation cows encounter the aforementioned management and physiological stressors for the first time (Vergara et al., 2014; Zebeli et al., 2015; Stauder et al., 2020). In addition, first-lactation cows are often naive to diets that contain high levels of rapidly fermentable carbohydrates and their rumen papillae are comparably shorter and less numerous than in multiparous cows (Penner et al., 2007; Humer et al., 2015; Zebeli et al., 2015).

Despite their generally higher risk to develop SARA, the severity in first-lactation cows has repeatedly been shown to largely vary between individuals, even when receiving the same diet (Humer et al., 2015; Nasrollahi et al., 2017; Khiaosa-Ard et al., 2018). Yet, the causes of different SARA severity remain scarcely understood and it is unclear whether rumen fermentation factors, animal behavior and metabolism or other mechanisms are the root cause (Nasrollahi et al., 2017; Khiaosa-Ard et al., 2018; Yang et al., 2022). In addition, the impact of SARA severity for cow health and behavior are not yet clear. Consequently, there is a substantial need for investigation in order to gain knowledge on the underlying mechanisms of SARA severity and its implications in dairy cows. This will be a prerequisite to design feeding strategies for preventing or alleviating the detrimental consequences of high-grain feeding-induced SARA in dairy cows, particularly in primiparous cows.

The objective of our study was to assess the SARA severity in first-lactation Holstein cows induced by feeding the same feeding regimen during transition and lactation, and to evaluate the factors that influence this severity, as well as to investigate the consequences of the different SARA severities on various parameters of animal behavior and health as well as rumen and hindgut fermentation in first-lactation Holstein cows. We hypothesized differences in behavior and health status as well as rumen and hindgut fermentation of first-lactation cows with different SARA severity and less ruminating activity as well as higher inflammation markers in susceptible cows than in cows resistant to SARA.

## Materials and Methods

The study was approved by the Institutional Ethics and Animal Welfare Committee of the University of Veterinary Medicine Vienna and the national authority according to §26 of the Law for Animal Experiments, Tierversuchsgesetz 2012-TVG (GZ: 2021-0.009.975).

### Animals, housing, and experimental design

The experiment was conducted in a longitudinal design from February to December 2021 at the dairy research station VetFarm in Pottenstein, Austria. Before designing the study, an a priori power analysis was performed to make sure the trial had enough animals within each group differing in SARA severity, with sufficient power to differentiate between cows with high and low SARA severity. The power analysis suggested that a statistical test with a 0.05 two-sided significant level would have 90% power to detect an intergroup (low SARA severity vs. high SARA severity) difference for the minimal detectable difference (δ) of ruminal pH of 30%, suggesting a group size of at least 7-8 animals. The δ of 30% between extreme SARA groups was established based on our previous observations of ruminal pH in which cows were also fed a 60% concentrate diet (Stauder et al., 2020). Based on these recommendations of the power analysis, we selected 24 animals for this trial, assuming to have 12 animals in each of the two extreme SARA severity groups.

Therefore, 24 pregnant Holstein heifers were selected for this experiment. To minimize the level of variation, apart from using the same breed and only primiparous cows, all selected heifers were kept together under the same conditions, receiving the same diets and treatments before entering the experiment. When enrolled in the study, the heifers were pregnant, three weeks before the expected calving date, and weighing 699 ± 81.5 kg BW and being 27.1 ± 2.39 months of age. Differences in BW and age among heifers were mainly because they were enrolled in the trial by expected calving date, which, based on the successful insemination, was different. Thus, the heifers were enrolled in a step-wise manner in the experiment. In specific, 15 heifers entered the experiment in February, followed by 3 other heifers in March, 2 heifers in April, 1 heifer in August, and 3 heifers in September. Each enrollment was considered a block in the later statistical analysis. During the experiment, heifers were housed in a free-stall barn equipped with 15 deep litter cubicles (2.6 m × 1.25 m, straw litter) and a deep litter area (15.7 m × 8.1 m, straw litter), and kept in the experiment from around 3 wk antepartum (ap) until 10 wk postpartum (pp). Cows were adapted to the experimental barn area, including the feeding troughs, 1 wk before the trial and continuously supervised during the trial. No feeding or health issues requiring veterinary interventions occurred during the experiment. All cows calved in the deep litter area without any stimulation, and the pre-calving signs were monitored via video-surveillance and in person. There were no complications during the parturitions and the cows were separated via a mobile fence after calving for sample collection and animal health measurements, and returned to the group within maximum 1.5 h pp.

### Diets and feeding

Three weeks prior to the expected parturition, all heifers were offered a close-up diet as total mixed ration (TMR) containing 68% forage and 32% concentrate on dry matter (DM) basis (Table 1), that was formulated to meet the requirements in energy and nutrients of pregnant heifers (GfE, 2001). After calving, the cows were transitioned to 60% concentrate allowance by feeding first the close-up diet plus gradually increased amounts of lactation concentrate over the first 7 d post-calving (Supplementary Fig. S1). Afterward, cows were offered a high-grain diet as TMR containing 40% forage and 60% lactation concentrate on DM basis (Table 1), until the end of the experiment at 10 wk pp. The TMR were prepared once daily with an automatic feeding system (Trioliet Triomatic T15, the Netherlands) and delivered twice daily at approximately 0830 and 1600 hours in animal-individual controlled feeding troughs that allowed the continuous recording of individual feed intake via electronic weighing scales and computer-regulated access gates (Insentec B.V., the Netherlands). The cows were adapted to the troughs for one week before the beginning of the experiment. The troughs were emptied and cleaned thoroughly before each morning feeding. The cows were fed ad libitum by allowing for around 10% feed refusals and cows had free access to water during the entire experiment.

**Table 1.** Ingredients, chemical composition, particle size fractions and physically effective fiber of diets fed to cows before and after parturition (stated as % of dry matter unless not stated otherwise)

	Diets	
Item	Close-up	Lactation
Ingredients		
Grass silage	38.0	24.0
Corn silage	30.0	16.0
Pelleted close-up concentrate^1^	32.0	0.00
Pelleted lactation concentrate^2^	0.00	60.0
Chemical composition		
Dry matter, % as fresh	46.6	48.8
Crude protein	15.2	16.7
Neutral detergent fiber	41.1	34.3
Acid detergent fiber	26.1	20.8
Starch	18.1	25.0
Non-fiber carbohydrates	35.7	40.2
Ether extract	2.29	2.29
Ash	7.10	6.94
Particle size distribution, % retained^3^		
Long	19.2	10.4
Medium	42.5	31.5
Short	35.3	51.3
Fine	2.98	6.83
Physical effectiveness factor	0.62	0.42
Physically effective NDF_>8_	25.4	14.4

^1^Composition on a DM basis: barley meal (62.4%), rapeseed meal (20.0%), corn meal (9.0%), soybean meal (4.0%), Biomin vital dry (3.5%; Biomin Holding GmbH, Getzersdorf, Austria), molasses (1.0%), magnesium oxide (0.1%).

^2^Composition on a DM basis: barley meal (61.5%), rapeseed meal (16.0%), corn meal (10.00%), soybean meal (8.0%), Biomin M 18 (2.8%; Biomin Holding GmbH, Getzersdorf, Austria), molasses (1.0%), limestone (0.6%), magnesium oxide (0.07%).

^3^Particle fractions determined with the Penn State Particle Separator according to Kononoff et al. (2003).

### Measurement of ruminal pH metrics

The ruminal pH was continuously measured using indwelling wireless ruminal pH sensors (pH Plus Bolus SX-1042A, smaXtec animal care GmbH, Graz, Austria) that have been validated before by Klevenhusen et al. (2014). In brief, pH data from the indwelling sensors were compared to pH data obtained via measurements with a pH electrode in manually collected ruminal fluid samples. The data sets were statistically assessed for precision and accuracy properties to ensure reproducibility of methods, showing that the indwelling wireless ruminal pH sensors can satisfactorily reflect the ruminal fluid pH. Each cow received its bolus one week before entering the trial and before administration, all sensors were calibrated in a pH 7.0 calibration buffer according to the manufacturer’s protocol and then immediately orally administered into the reticulo-rumen of the cows using a bolus gun provided by the company. The pH was recorded in 10 min intervals, and various metrics of ruminal pH were measured such as daily minimum, maximum, variation and average of ruminal pH, as well as the duration and area under the curve (AUC) of ruminal pH < 5.8. Moreover, using the DM intake (DMI) data set, the SARA-index was calculated by relating the daily duration of ruminal pH < 5.8 to the daily DMI (Rivera-Chacon et al., 2022a).

### Ruminal pH metrics to characterize SARA and definition of SARA severity type

To differentiate the level of SARA severity in cows, we performed a cluster analysis by taking into account several metrics of the ruminal pH responses of the cows during the entire experiment. First, we calculated the days that each cow experienced SARA, using the duration of ruminal pH below 5.8 for longer than 330 min/d as a SARA threshold (Zebeli et al., 2008), and then related this number to the total experiment duration of each cow, thus accounting for the small differences in experiment length between individuals. Then, to fully characterize the ruminal pH responses and the SARA risk, we calculated various ruminal pH metrics such as minimum ruminal pH, maximum ruminal pH, ruminal pH variation, daily mean ruminal pH, duration of ruminal pH < 5.8, area of ruminal pH < 5.8, and the SARA index for the entire experiment. All these metrics then fed the matrix of the cluster analysis that employed both the PROC DISTANCE and PROC CLUSTER of SAS v9.4 (SAS Institute Inc., Cary, NC). A distance matrix based on Euclidian distances was designed with all the above-mentioned rumen pH metrics. The proximity measure was computed using the respective standard deviation of each variable and subsequently a Ward’s minimum-variance cluster analysis was performed using the already created distance matrix. The heights of the dendrogram were specified using the R-square method.

### Measurement of chewing, feed particle sorting, and lying behavior

The chewing behavior was measured during 3 consecutive days in week 3 ap, week 1 ap, and week 7 pp from 00:00 h to 23:59 h using validated rumination halters (RumiWatch System, ITIN + Hoch GmbH, Switzerland; Kröger et al., 2016) according to the procedure and the parameters described by Rivera-Chacon et al. (2022b). In brief, all halters were attached to the cows one day before the measurements started to allow the cows to get used to them. Furthermore, the correct position of the halters as well as the proper recording of the data were checked twice daily. Consequently, chewing parameters related to eating and ruminating were determined. The feed particle sorting behavior was analyzed as described by Haselmann et al. (2019). In week 1 ap, week 5 pp, and week 10 pp, offered TMR and the orts were collected from each feeding trough of individual cows in the morning of the following day, respectively. The particle size distribution was determined using a Penn State Particle Separator (model C24682N, Nasco, ID) following the method of Kononoff et al. (2003). Then, the proportional sorting indices were calculated for each particle fraction on an as-fed basis by relating the amount of each fraction in the TMR orts to the amount of each fraction in the offered TMR. Consequently, a sorting index of 100 indicated no sorting of the particle fraction, whereas a sorting index above indicated that the cows sorted for that fraction and a sorting index below 100 indicated that the cows sorted against that fraction. The diurnal lying behavior of cows was determined on all days in week 2 pp and week 7 pp using data loggers (HOBO Pendant G Acceleration Data Logger, Onset Computer Corp., Bourne, MA) and following the protocol and parameters outlined by Rivera-Chacon et al. (2022b).

### Sample collections

Except for blood samples, all samplings were conducted in a weekly pattern. The feed samples were collected at 3-wk intervals during the complete trial. Thereby, individual feedstuffs, i.e., grass silage, corn silage, and concentrates, were taken from feed bunkers and TMR samples were taken directly from the automatic feeding system after preparation in the morning. All feed samples were directly stored at −20 °C until further analysis.

Ruminal fluid was collected before morning feeding from each cow in week 3 ap, week 2 pp, week 4 pp, and week 8 pp using a stomach tube with the method described by Steiner et al. (2015). After discarding the first 100 mL, several sample aliquots were taken using a sterile disposable syringe and subsequently stored at −20 °C until further analysis.

The fecal sampling was performed as outlined by Castillo-Lopez et al. (2020) before morning feeding in a biweekly interval, i.e., in week 3 ap, week 1 ap, week 2 pp, week 4 pp, week 6 pp, week 8 pp, and week10 pp. Immediately after collection, the fecal pH was measured using a portable pH meter (Mettler-Toledo AG Analytical, Schwerzenbach, Switzerland) by direct insertion of the pH sensor into the fecal sample. Afterward, sample aliquots were taken and frozen at−20 °C until further analysis.

The blood sampling took place before the morning feeding in 12 wk during the experiment, namely week 3 ap, week 1 ap, day 1 pp, day 3 pp, day 7 pp, day 10 pp, week 2 pp, week 3 pp, week 4 pp, week 6 pp, week 8 pp, and week 10 pp. For each blood collection, cows were fixed in the headlock and blood was taken from the jugular vein using evacuated tubes for serum collection (9 mL, Vacuette, Greiner Bio-One, Kremsmuenster, Austria). The tubes were allowed to clot for 1.5 h at room temperature and subsequently centrifuged at 2,000 × *g* for 15 min at 4 °C (Centrifuge 5804 R, Eppendorf, Hamburg, Germany). Then, serum aliquots were stored at −80 °C until further analysis.

### Analysis of feed samples

The feed samples were dried at 65 °C in a forced-air oven for 48 h and ground through a 1-mm screen (Ultra Centrifugal Mill ZM 200, Retsch, Haan, Germany) before they were analyzed in accordance with the Association of German Agricultural Analytic and Research Institutes (VDLUFA, 2012) for DM (method 3.1), ash (method 8.1), crude protein (method 4.1.1), and ether extract (method 5.1.2). The neutral detergent fiber assayed with a heat stable α-amylase and expressed exclusive of residual ash (aNDFom; method 6.5.1) and acid detergent fiber expressed exclusive of residual ash (ADFom; method 6.5.2) were determined using the Fibretherm FT 12 (Gerhardt GmbH & Co. KG, Königswinter, Germany). The total starch content was determined using a commercially available kit (Megazyme, Wicklow, Ireland). Physically effective NDF larger than 8 mm (peNDF_>8_) was measured after analyzing the particle size of the diets and orts, using the Penn State Particle Separator (model C24682N, Nasco; Kononoff et al., 2003). Therefore, weight of particles retained on the sieves with a screen ≥ 8 mm was multiplied with the NDF concentration in the diet or orts.

### Analysis of ruminal fluid and fecal samples

The volatile fatty acid (VFA) profiles were determined by gas chromatography using the procedure described by Hartinger et al. (2022). For the fecal samples, sample processing before the first centrifugation step was slightly modified; 1 g feces was diluted in 1 mL distilled water and subsequently mixed with 300 µL of the internal standard and 200 µL of 25% phosphoric acid (Castillo-Lopez et al., 2020). Additionally, ruminal fluid aliquots were further analyzed for ammonia-N concentration using an indophenol colorimetric method on a U3000 spectrophotometer (INULA GmbH, Vienna, Austria).

### Analysis of blood variables

The concentrations of the liver enzymes aspartate aminotransferase (AST), γ-glutamyl-transferase (GGT), and glutamate dehydrogenase (GLDH) were analyzed in duplicate in blood sera using standard enzymatic colorimetric assays. The analyses were performed on a fully automated analyzer for clinical chemistry (Cobas 6000/c501; Roche Diagnostics GmbH, Austria) at the laboratory of the Central Clinical Pathology Unit (University of Veterinary Medicine, Vienna, Austria) and the intra-assay variation for all blood chemistry assays was ≤ 5%. The serum concentrations of the acute-phase proteins (APP), i.e., haptoglobin and serum amyloid A (SAA), were measured with a commercially available cow ELISA kit (Life Diagnostics Inc., West Chester, PA) and a commercially multispecies ELISA kit (Tridelta, Maynooth, Ireland), respectively, on a spectrophotometer (xMark Microplate Absorbance Spectrophotometer; Bio-Rad Laboratories GesmbH, Vienna, Austria) following the manufacturer’s protocols.

### Statistical analyses

The data sets were analyzed in SAS v9.4 (SAS Institute Inc.) using the Shapiro-Wilk’s normality method of PROC UNIVARIATE to validate normal distribution. If the data of a certain variable were not normally distributed, they were logarithmically or in a second step square-root transformed. Afterward, an ANOVA was performed using PROC MIXED with the following model:


$$\begin{array}{l} Y_{ijk}=\mu +s_{i}+t_{j}+{\left(s\times t\right)}_{ij}+c_{k}+e_{ijk} \end{array}$$

where µ is the mean, *s*_*j*_ is the fixed effect of SARA type, *t*_*j*_ is the fixed effect of time (depending on variable, experimental week or day), *c*_*k*_ is the random effect of individual cow within block, and *e*_*ijk*_ is the residual error. As the cows were enrolled on different months and the different ambient temperature might affect SARA (Mishra et al., 1970), we initially checked the role of ambient temperature when cows entered into the trial by using this variable as a covariate on SARA duration, AUC, and SARA index. Since the analysis showed no significant effect of the covariate, i.e., *P* = 0.81, *P* = 0.80, and *P* = 0.67 for SARA duration, AUC, and SARA index, respectively, this was not further considered in the model. Measurements obtained on the same cow but at different times were considered as repeated measurements. Cow was used as the experimental unit. The least square means were post hoc tested by Tukey’s test. The significance level was defined at *P* ≤ 0.05 and tendencies were declared at 0.05 < *P* < 0.10 for all statistical analyses.

The odds of various factors of SARA severity were evaluated with the PROC LOGISTIC of SAS (version 9.4, SAS Institute Inc.). A model with a forward selection method was used to evaluate all potential influencing factors, by selecting only those showing a significant effect (*P* < 0.05). We evaluated the influence of several factors including the exact duration of close-up feeding, age of heifers at calving, DMI, starch intake, peNDF intake, as well as the variables of chewing behavior, feed particle sorting behavior, rumen fermentation variables (total and individual VFA). We further computed the odds ratios (OR) and respective profile likelihood CI of variables causing the event of HIGH SARA severity, as contrasted by LOW SARA severity. Due to the limited size of our data set, we omitted the additional investigation of the influential factors for MOD SARA severity. The ‘units’ statement was applied to specify units of change for the continuous explanatory variables. The analysis of maximum likelihood estimates was performed to evaluate the effects of continuous explanatory variables (i.e., Wald chi-square test), compute the Wald CI and the predicted probability of SARA occurrence (i.e., using the option PLOTS = EFFECTS). The receiver operating characteristic curve with the AUC were also computed.

## RESULTS

### Ruminal pH metrics and SARA indicators

Instead of the presumed two, the cluster analysis revealed three clearly discernable clusters of ruminal pH metric responses (i.e., SARA severities), which we termed as SARA-types (Supplementary Fig. S2). The first clearly discernable cohort of cows with similarity R-squared-index of > 80% was the cluster with 6 cows with low SARA severity (termed LOW). The LOW cows exceeded the SARA threshold of a ruminal pH < 5.8 for longer than 330 min/d up to a maximum of 7% of the experimental days. A second cluster grouped 9 cows together (R-squared similarity index of around 75%) with a moderate SARA severity (termed MOD). These 9 MOD cows exceeded the defined SARA threshold between 9% and 24% of the experimental days. As indicated by the cluster analysis, the LOW and MOD groups had a similarity index of 65% among them. Further, the cluster analysis clearly clustered a third cohort of 9 cows being highly susceptible to SARA (termed HIGH). The cluster analysis revealed that the latter group was clearly discernable from LOW and MOD SARA-types with a similarity index of almost 0% regarding the ruminal pH responses. The 9 HIGH cows exceeded the SARA threshold between 20 - 87% of the experimental days.

**Figure 1.**
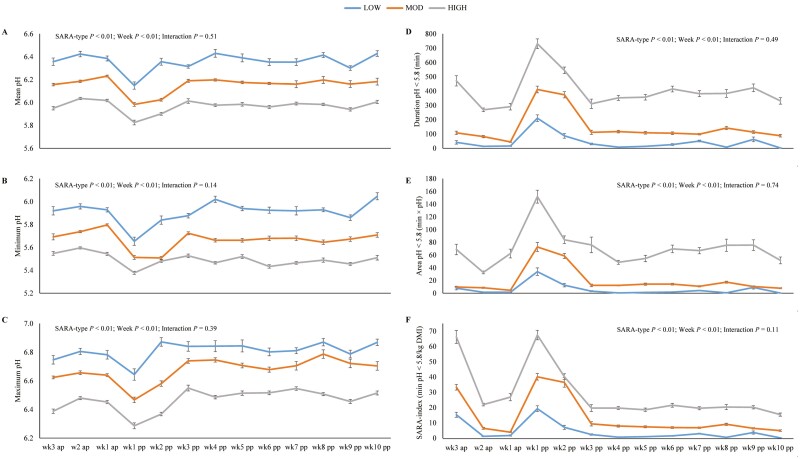
Ruminal daily mean pH [A], daily minimum pH [B], daily maximum pH [C], as well as daily duration of ruminal pH < 5.8 [D], area under the curve of ruminal pH < 5.8 [E] and SARA-index, calculated by relating the daily duration of ruminal pH < 5.8 to the daily DMI [F] of first-lactation Holstein cows with low (LOW, blue), moderate (MOD; orange), and high (HIGH, grey) SARA severity in different weeks before (ap) and after parturition (pp). Lines illustrate least square means with respective standard errors.

The ANOVA showed that the HIGH cows had the lowest daily mean pH, ranging between pH 5.83-6.04, followed by MOD cows with a daily mean pH range of pH 5.98-6.23 and LOW cows with the highest daily mean pH of 6.15-6.43 (Fig. 1A). Likewise, HIGH cows had lowest minimum and maximum pH in the rumen (Fig. 1B-1C) of all SARA-types. The duration and AUC of ruminal pH < 5.8 of HIGH cows were many times higher than of MOD and LOW cows, respectively (Fig. 1D-1E). The SARA-index was highest for HIGH cows as well, and approximately 2- and 8-fold higher than for MOD and LOW cows (Fig. 1F). Regarding the effect of week relative to parturition, ruminal pH variables were mainly affected in the first 2 weeks after parturition (each *P* < 0.01). Supplementary Fig. S3 illustrates the diurnal ruminal pH pattern around parturition, where HIGH cows showed a clear pH drop on the day of calving that set in around 24 h in advance. This pattern was also present, but less distinctive in MOD cows, whereas LOW cows had no reduction in ruminal pH around calving.

### Factors influencing the likelihood for high SARA severity

**Figure 2.**
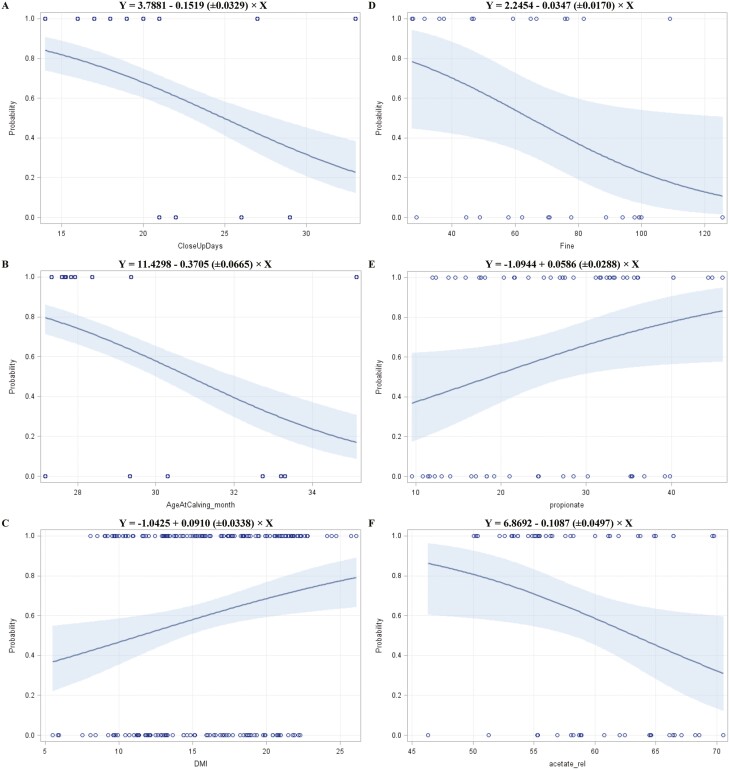
The predicted probability of SARA severity in first-lactation Holstein cows with the 95% confidence limits (shaded area) based on the duration of close-up feeding [A], the age at parturition [B], dry matter intake [C], sorting index of fine particles [D], absolute propionate concentration in the rumen [E], and relative acetate concentration in the rumen [F].

The logistic analysis suggested 6 variables as influencing factors of high SARA severity. Accordingly, the analysis of maximum likelihood estimates and the Wald chi-square test predicted a reduced likelihood of cows becoming a HIGH SARA-type with each additional day of close-up feeding (*P =* 0.01; Fig. 2A). Thereby, 1 wk longer of close-up feeding reduced the odds of becoming a HIGH SARA-type by 34.5% (Wald 95% CI: 0.22-0.53). The age at parturition affected the SARA severity (*P* < 0.01; Fig. 2B) in a way that the odds of cows becoming a HIGH SARA-type was reduced by 69.0% (Wald 95% CI: 0.60-0.78) when being one month older at calving. Other findings were that DMI was an influencing factor and predicted to increase the risk for high SARA severity (*P =* 0.01; Fig. 2C) with an OR of 1.10 (Wald 95% CI: 1.03–1.17) for every additional kg DMI. The SARA likelihood was reduced when cows sorted for fine particles (*P =* 0.04; Fig. 2D) with a predicted OR of 0.71 (Wald 95% CI: 0.49–0.96) for a 10% lower sorting index. The absolute propionate concentration in the rumen was suggested to be an influencing factor (*P* = 0.04; Fig. 2E), so that 2 m*M* more of this VFA were predicted to increase the OR to 1.12 (Wald 95% Cl: 1.01-1.27). The relative acetate concentration in the rumen was predicted to reduce the likelihood of cows becoming a HIGH SARA-type by 19.5% (Wald 95% Cl: 0.64-0.97) per 2 percentage points increase in ruminal acetate proportion (*P* = 0.03; Fig. 2F). Other variables analyzed showed a *P*-value > 0.05.

### Feed intake

**Figure 3.**
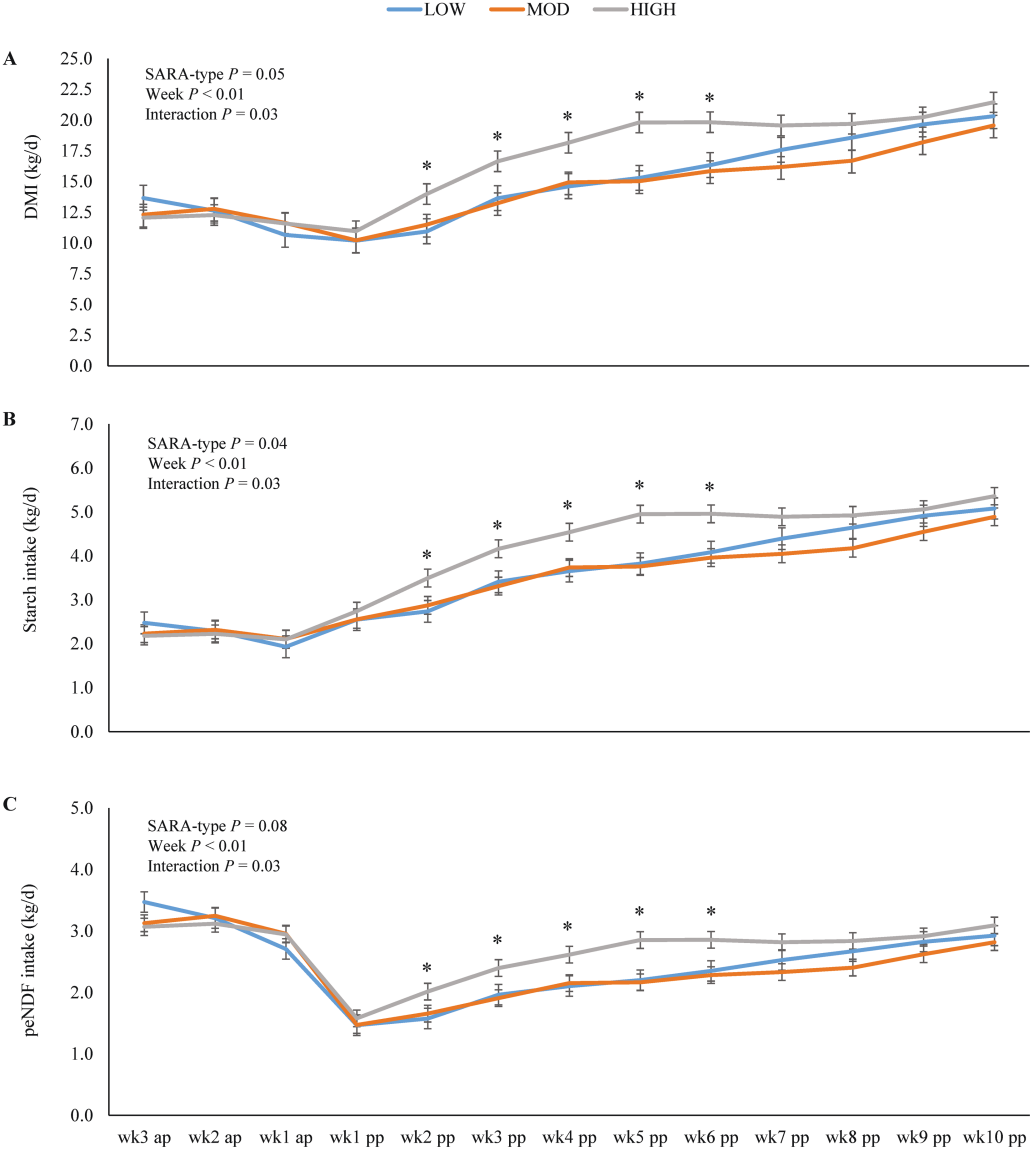
Daily intake of dry matter (DMI) [A], starch [B], and physically effective neutral detergent fiber (peNDF_>8_) [C] of first-lactation Holstein cows with low (LOW, blue), moderate (MOD; orange), and high (HIGH, grey) SARA severity at in weeks before (ap) and after parturition (pp). Lines illustrate least square means with respective standard errors. Asterisks indicate significant difference between SARA-types within an experimental week (*P* < 0.05).

We observed an interaction of SARA-type and week relative to parturition (*P* = 0.03) with higher DMI of HIGH than of MOD and LOW cows from week 2 pp until week 6 pp (Fig. 3A). Regarding the starch intake (Fig. 3B), the interaction of SARA-type and week relative to parturition again showed higher starch intake by HIGH cows compared to MOD and LOW cows from week 2 pp until week 6 pp (*P* = 0.03). We also found an interaction of SARA-type and week relative to parturition for peNDF intake (Fig. 3C; *P* = 0.05) with higher intakes of HIGH than of MOD and LOW cows during a 4-week period from week 2 pp until week 6 pp. In contrast to starch intake, the SARA-type, as a main effect, showed no statistically significant impact on peNDF intake (*P* = 0.08).

### Chewing, feed sorting, and lying behavior

Regarding the chewing behavior (Table 2), we found an interaction of SARA-type and week relative to parturition for total ruminating time (*P* < 0.01), ruminating time related to DMI (*P* = 0.01), total ruminating chews per day (*P* < 0.01), and per bolus (*P* = 0.03). Hereby, total rumination time and total ruminating chews per day of HIGH cows was lower in week 1 ap compared to week 3 ap and week 7 ap, while when expressed per unit of DMI, HIGH cows ruminated around 16 min less per kilogram DM in week 3 ap than in week 7 pp, with week 1 ap being in between. Likewise, chewing index was lower for HIGH cows in week 7 pp when compared to week 3 ap with week 1 ap as intermediate, while the post hoc test revealed no least square mean differences for ruminating chews per bolus. Moreover, the SARA-type affected the absolute eating time per day (*P = *0.03). The week relative to parturition affected the numbers of ruminating chews and ruminating boli per day (both *P* < 0.01), which were higher in week 3 ap and week 7 pp than in week 1 ap. Furthermore, ruminating chews per min were higher in week 7 pp than in week 3 ap and week 1 ap (*P* < 0.01). In contrast, absolute daily eating time was approximately an hour less before than after parturition, i.e., lower in week 3 ap and week 1 ap than in week 7 pp (*P* = 0.01).

**Table 2.** Chewing activity in first-lactation Holstein cows during different weeks relative to parturition and differing in SARA severity^1^

Variable	Week 3 ap^2^			Week1 ap			Week7 pp^3^			SEM	*P*-values		
	LOW	MOD	HIGH	LOW	MOD	HIGH	LOW	MOD	HIGH		SARA-type	Week	Interaction
Rumination, min/d	419	511	488^A^	406	339	352^B^	513	521	489^A^	28.8	0.78	<0.01	<0.01
Rumination, min/kg DMI	31.7	41.9	41.8^A^	38.5	30.2	30.3^AB^	31.0	33.3	25.4^B^	3.97	0.63	<0.01	0.01
Eating, min/d	281	209	251	270	199	246	351	290	282	35.3	0.04	0.01	0.79
Eating, min/kg DMI	21.3	16.9	19.6	21.8	17.5	19.8	20.5	18.4	14.9	2.52	0.12	0.53	0.39
Ruminating chews, n/d	27,425	33,164	33,200^A^	28,258	22,222	23,303^B^	32,788	34,550	31,947^A^	2144	0.95	<0.01	<0.01
Ruminated boli, n/d	448	583	514	431	381	378	532	547	508	39.0	0.34	<0.01	0.09
Ruminating chews, n/min	74.5	75.0	74.6	75.7	73.6	72.9	70.6	72.1	71.1	1.59	0.87	<0.01	0.33
Ruminating chews, n/bolus	61.2	62.0	62.6	64.2	57.4	60.3	60.9	61.7	60.8	2.07	0.69	0.42	0.03
Chewing index^4^	53.0	58.8	61.4^A^	65.0	47.0	49.6^AB^	51.7	51.7	40.3^B^	6.01	0.26	0.03	0.03

^1^SARA severity based on a cluster analysis considering rumen pH metrics and resulted in 3 distinct severities, i.e., low (LOW), moderate (MOD), and high (HIGH) SARA severity.

^2^Antepartum.

^3^Postpartum.

^4^Sum of ruminating and eating time divided by dry matter intake (min/kg DMI).

Capitalized superscript letters indicate differences between weeks within the same SARA type (*P < *0.05).

The results of feed particle sorting behavior analysis are given in Table 3. The interaction of SARA-type and week relative to parturition affected the selection index of peNDF (*P* < 0.01) with more sorting for peNDF by MOD cows in week 5 than in week 1 and week 10. The SARA type did not influence the feed particle sorting behavior of the cows (each *P > *0.05), but LOW cows tended to select to a lower extent against fine particles (*P* = 0.09). Furthermore, the selection index for long particles was affected by week relative to parturition (*P *=* *0.01) and lower in week 1 ap and week 10 pp than in week 5 pp.

**Table 3.** Feed particle sorting behavior, presented as selection indices on as-fed basis^1^, in first-lactation Holstein during various weeks relative to calving and differing in SARA severity^2^

Variable	Week 1 ap^3^			Week 5 pp^4^			Week 10 pp				*P*-values		
	LOW	MOD	HIGH	LOW	MOD	HIGH	LOW	MOD	HIGH	SEM	SARA-type	Week	Interaction
Long particle size	102	94.1	78.1	97.3	116	107	96.5	90.1	87.6	9.55	0.50	0.01	0.10
Medium particle size	104	103	108	91.9	106	104	105	107	103	4.31	0.23	0.25	0.21
Short particle size	100	104	105	104	102	101	102	103	104	2.11	0.87	0.57	0.13
Fine particle size	81.5	59.7	55.0	75.2	58.7	48.2	79.0	48.7	75.3	15.14	0.09	0.76	0.67
peNDF^5^	104	100^B^	102	97.7	112^A^	106	105	107^AB^	102	4.02	0.52	0.30	<0.01

^1^Sorting index of 100 indicates no sorting of a particle fraction, sorting index above 100 indicates sorting for a particle fraction, and sorting index below 100 indicates sorting against a particle fraction.

^2^SARA severity based on a cluster analysis considering rumen pH metrics and resulted in 3 distinct severities, i.e., low (LOW), moderate (MOD), and high (HIGH) SARA severity.

^3^Antepartum.

^4^Postpartum.

^5^Physically effective neutral detergent fiber.

Capitalized superscript letters indicate differences between weeks within the same SARA-type (*P < *0.05).

The SARA-type did not affect the variables of lying behavior (each *P* > 0.10; Table 4). The week relative to parturition influenced the number of total daily lying bouts (*P *=* *0.03) as well as the daily lying time on the left side (*P < *0.01) and lying bout on left side (*P < *0.01). Cows showed a longer daily lying time (*P < *0.01) and number of daily lying bouts on the right side (*P < *0.01), as well as durations of lying bouts on left and right sides (both *P < *0.01) in week 7 pp than in week 2 pp. No interaction of SARA type and week relative to parturition was found for lying behavior (each *P ≥ *0.05), but daily lying bouts on the left side tended to be lower in week 7 pp than in week 2 pp for MOD and HIGH cows, but not LOW cows (*P* = 0.06).

**Table 4.** Effects of SARA-type and week relative to parturition on lying behavior in first-lactation Holstein cows with different SARA severity^1^

Variable	Week 2 pp^2^			Week 7 pp				*P*-values		
	LOW	MOD	HIGH	LOW	MOD	HIGH	SEM	SARA-type	Week	Interaction
Total standing time, h/d	13.6	13.5	13.7	13.4	13.9	13.6	0.58	0.95	0.88	0.30
Total lying time, h/d	10.4	10.5	10.3	10.6	10.1	10.4	0.58	0.95	0.88	0.30
Lying time right side, h/d	0.85	0.72	0.94	1.47	1.22	1.84	0.50	0.74	<0.01	0.31
Lying time left side, h/d	9.79	9.83	9.34	9.39	8.98	8.64	0.69	0.76	<0.01	0.65
Duration of each lying bout right side, h	0.10	0.10	0.12	0.15	0.16	0.16	0.10	0.88	<0.01	0.89
Duration of each lying bout left side, h	0.50	0.44	0.50	0.55	0.50	0.53	0.05	0.64	<0.01	0.48
Total lying bouts, n/d	22.8	26.9	23.2	22.1	22.4	22.7	0.25	0.73	0.03	0.10
Lying bouts right side, n/d	3.73	4.93	4.87	5.66	5.40	6.96	1.45	0.78	<0.01	0.26
Lying bouts left side, n/d	19.7	22.7	19.1	17.4	17.8	16.6	1.58	0.37	<0.01	0.06

^1^SARA severity based on a cluster analysis considering rumen pH metrics and resulted in 3 distinct severities, i.e., low (LOW), moderate (MOD), and high (HIGH) SARA severity.

^2^Postpartum.

### Ruminal and fecal fermentation profile

The HIGH cows tended to have higher total VFA concentrations in the rumen than MOD cows with LOW cows having intermediate values (*P* = 0.09; Table 5). Regarding the VFA profile, HIGH and MOD cows had lower acetate proportions than LOW cows (*P* = 0.01). The propionate proportions were higher in HIGH cows than in LOW cows with MOD as intermediate (*P* = 0.05). The ratios of acetate to propionate or lipogenic VFA to glucogenic VFA were affected by SARA-type (both *P* = 0.03) and were both lower in HIGH cows than in LOW cows with MOD cows as intermediate. The ruminal ammonia-N concentration was not affected by SARA but we observed a main effect of week relative to calving with higher ammonia-N concentration in week 8 pp compared to week 3 ap and week 2 pp (*P* < 0.01; Supplementary Fig. S4).

**Table 5.** Ruminal volatile fatty acid (VFA) profile in first-lactation Holstein cows on different weeks relative to parturition and differing in SARA severity^1^

	Week 3 ap^2^			Week 2 pp^3^			Week 4 pp			Week 8 pp				*P*-values		
	LOW	MOD	HIGH	LOW	MOD	HIGH	LOW	MOD	HIGH	LOW	MOD	HIGH	SEM	SARA-type	Week	Interaction
Total VFA, µmol/mL	70.4	65.1	83.3	83.4	79.5	93.8	93.7	80.8	98.8	92.1	101	102	8.06	0.09	<0.01	0.69
Acetate, %	67.2	67.5	65.2	58.3	53.5	53.4	59.9	57.8	56.5	59.5	54.3	56.0	1.57	0.01	<0.01	0.41
Propionate, %	18.5	17.6	20.1	28.5	30.3	32.5	28.6	29.7	31.9	28.7	34.6	33.2	1.81	0.05	<0.01	0.50
n-Butyrate, %	10.2	11.0	10.5	9.25	12.1	10.4	7.26	7.64	7.46	7.36	6.50	6.31	0.66	0.24	<0.01	0.11
n-Valerate, %	1.19	1.17	1.36	1.59	1.77	1.74	1.62	2.15	1.87	1.57	2.24	1.85	0.18	0.09	<0.01	0.41
iso-Butyrate, %	1.05	1.03	0.97	0.74	0.59	0.57	0.84	0.77	0.76	0.83	0.62	0.73	0.06	0.04	<0.01	0.60
iso-Valerate, %	1.46	1.38	1.44	1.19	1.49	1.12	1.37	1.39	1.13	1.34	1.21	1.36	0.16	0.52	0.55	0.42
Caproate, %	0.44	0.42	0.46	0.38	0.30	0.29	0.43	0.52	0.29	0.64	0.42	0.50	0.13	0.66	0.25	0.71
Heptonate, %	0.00	0.00	0.00	0.00	0.00	0.00	0.03	0.08	0.03	0.13	0.09	0.06	0.04	0.78	0.02	0.85
Acetate:Propionate^4^	3.68	3.84	3.33	2.21	1.91	1.68	2.01	2.21	1.80	2.19	1.58	1.72	0.21	0.03	<0.01	0.55
Lipogenic:Glucogenic^5^	3.98	4.19	3.61	2.40	2.21	1.91	2.35	2.11	1.93	2.33	1.66	1.82	0.23	0.03	<0.01	0.50

^1^SARA severity based on a cluster analysis considering rumen pH metrics and resulted in 3 distinct severities, i.e., low (LOW), moderate (MOD), and high (HIGH) SARA severity.

^2^Antepartum.

^3^Postpartum.

^4^Ratio of acetate (µmol/mL) to propionate (µmol/mL).

^5^Ratio of the sum of acetate (µmol/mL) and n-butyrate (µmol/mL) to the sum of propionate (µmol/mL) and *n*-valerate (µmol/mL).

The fecal VFA and the related VFA profiles were not different between the 3 SARA types (Supplementary Table S1). However, we observed an interaction of SARA type and week relative to parturition for the fecal iso-valerate (*P < *0.01) with higher proportions in LOW cows than in MOD cows during week 3 ap, as well as higher proportions in LOW cows in week 3 ap than in week 2 pp and week 8 pp. Similarly, interactions were present for the fecal proportions of acetate (*P < *0.01) and *n*-valerate (*P = *0.02). The fecal pH of the cows varied between 6.5 and 6.9 and was not affected by SARA type, week relative to parturition, or their interaction (each *P* > 0.10; Supplementary Fig. S5).

### Blood variables

**Figure 4.**
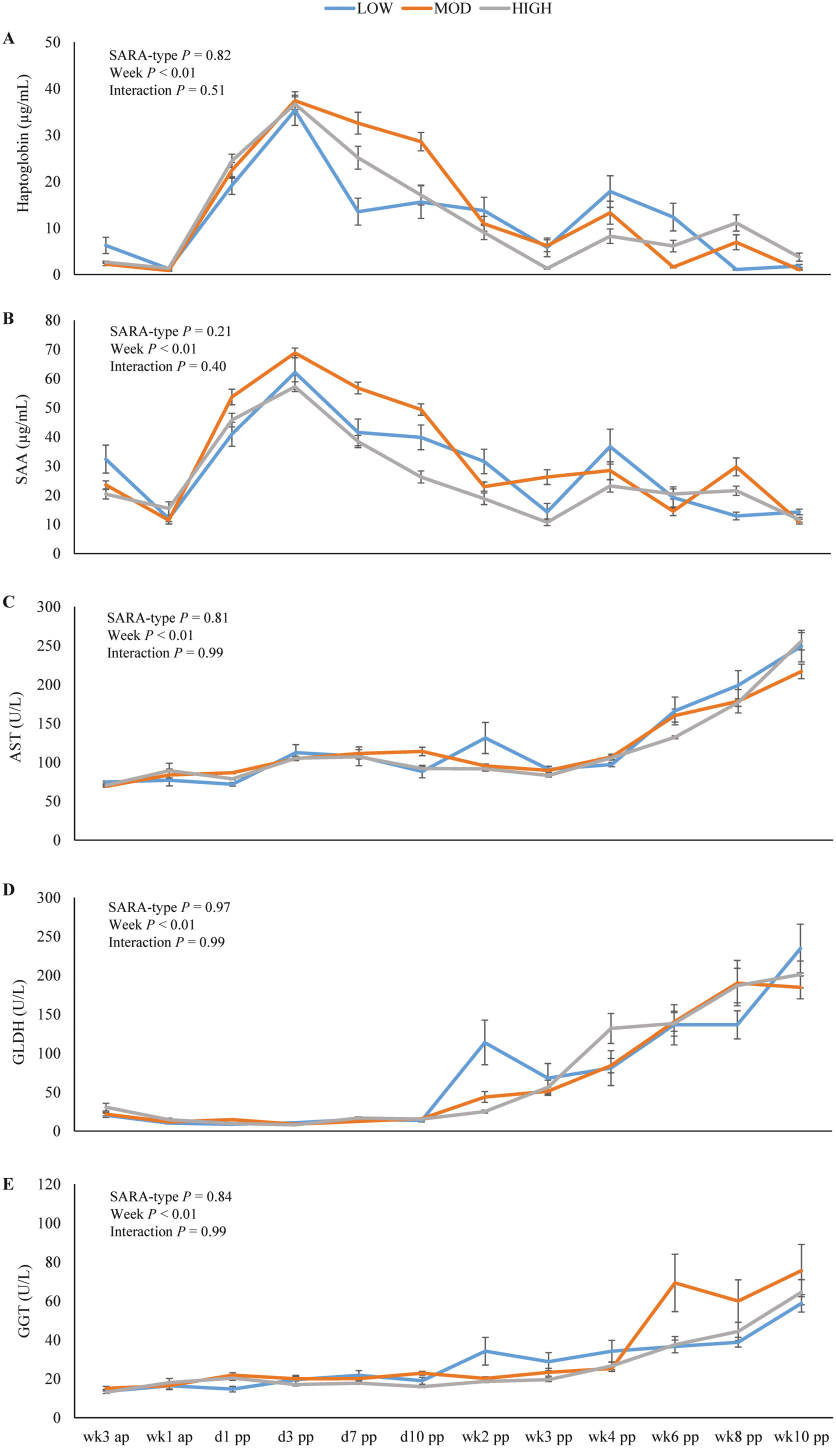
Serum concentrations of acute-phase proteins haptoglobin [A], serum amyloid A (SAA) [B] as well as liver enzymes aspartate aminotransferase (AST) [C], glutamate dehydrogenase (GLDH) [D], and γ-glutamyl-transferase (GGT) [E] of first-lactation Holstein cows with low (LOW, blue), moderate (MOD; orange), and high (HIGH, grey) SARA severity in different weeks before (ap) and after parturition (pp). Lines illustrate least square means with respective standard errors.

The concentrations of haptoglobin and SAA were not affected by SARA type (both *P > *0.10; Fig. 4A-4B). In contrast, the concentrations of both APP increased after calving and were higher from day 1 pp until day 10 pp compared to all later weeks or weeks before parturition (each *P < *0.01). We found no differences between cows with different SARA type for liver enzyme concentrations (each *P > *0.10; Fig. 4C-4E). However, the liver enzymes were affected by week relative to parturition (each *P < *0.01) with higher concentrations of AST and GLDH from wk6 pp on when compared to all weeks before as well as higher GGT concentrations in week 10 pp compared to all weeks before week 4 pp.

## Discussion

The first goal of this study was to assess the SARA severity in first-lactation Holstein cows induced by feeding the same feeding regimen during transition and early lactation period. The cluster analysis confirmed the presence of 3 clearly distinct SARA severities. When using the daily mean ruminal pH of 6.16 as a SARA threshold (Zebeli et al., 2008), HIGH cows experienced SARA during the entire experiment, while LOW cows undercut a mean ruminal pH of 6.16 only during the first week of high-grain feeding, i.e., week 1 pp. Likewise, using the more commonly applied SARA definition of ruminal pH < 5.8 for > 330 min/d (Zebeli et al., 2008), HIGH cows had SARA for 77% of the experimental period, while LOW cows only in maximum 7% of the experimental days. Varying SARA severities have also been identified in multiparous Holstein cows (Yang et al., 2022), showing that this is a common phenomenon in dairy cattle. However, the SARA intervals of cows with high severity were substantially longer in our study than by Yang et al. (2022), which may be due to the fact that primiparous cows are more prone to SARA than multiparous cows (Stauder et al., 2020) and emphasizes the cows’ vulnerability during the first lactation. Unexpectedly, cows within each group experienced SARA with almost similar duration throughout the complete experiment, indicating to be rather independent of moderate- or high-grain feeding and feed intake level. In addition to the clearly increased SARA duration, HIGH cows also suffered from explicitly increased SARA intensity as indicated by multiple higher AUC than LOW cows. Likewise, also MOD cows experienced less lasting and severe SARA than HIGH cows and were more closely clustered to LOW cows. Yet, previous research has established that first-lactation cows typically suffer from higher SARA prevalence than multiparous cows as they are naive to energy-dense lactation diets and encounter lactation-related stressors for the first time (Humer et al., 2015; Zebeli et al., 2015). Our study provides clear evidence for a high variation of SARA severity also within primiparous cows, ranging from the LOW to the HIGH SARA-type.

Another main aim of this research was to evaluate the factors that influence the severity of SARA in first-lactation Holstein cow. It is commonly known that certain factors trigger SARA in dairy cows, such as parity, high starch inclusion, low peNDF level, and abrupt changes in the diet (Humer et al., 2018; Khorrami et al., 2021). And indeed, based on the recommendations by Khorrami et al. (2021), with 14.4% peNDF_>8_ and 25% starch the lactation diet of our study can be considered as a risky diet to induce SARA. However, the influence of such dietary factors was eliminated in our study as all cows were continuously exposed to the same feeding regimen and housing conditions. It is therefore among the most crucial concerns to determine the factors that were driving these clearly divergent SARA types. The logistic analysis revealed that the duration of close-up feeding was a strong factor for high SARA severity. Humer et al. (2018) had emphasized the importance of a close-up period to prepare the cows for lactation diets to prevent SARA. Yet, these authors did not specify any optimal duration, and others have suggested about 3 weeks or even shorter (Beever, 2006). Considering our results, however, it seems that longer close-up feeding periods of at least 4 weeks are necessary to efficiently diminish a high SARA severity of SARA during early lactation in primiparous cows. This effect of the length of close-up feeding on SARA severity may be explained with the ruminal absorptive capacity and rumen papillae development of heifers (Penner et al., 2007; Dieho et al., 2016). Hence, it seems logical that the strongest acidotic insults occurred in the first 2 wk after calving. Age at parturition was a second substantial influencing factor for high SARA severity. The common practice is to introduce heifers early in reproduction, resulting in a recommended age at first calving of 22-26 mo (Sawa et al., 2019), which is considerably lower than in our study where cows had a first calving age of on average 28.7 ± 2.74 months. Our data indicate that such breeding strategy may predispose primiparous cows to high SARA in the lactation, which has to be pondered carefully against potential increments in lifetime milk production. Consequently, it was revealed that close-up feeding and age at parturition are two important heifer management factors to be taken into consideration in the prevention of SARA.

The fact that high DMI increased the risk for high SARA severity is in line with prior research (Zebeli et al., 2008; Khorrami et al., 2021), and indicates that higher DMI favor fermentative processes rather than buffering processes in the rumen. This can be explained by the fact that the higher the DMI the higher the starch intake, meaning a higher production of VFA in the rumen. While the buffering capacity of the concomitantly increased peNDF intake is considered constant, the acidotic potential from the increased starch-derived VFA production is not, but increases linearly and therefore exceeds the ruminal buffering capacity (Shaver, 2002). The point at which this balance between concomitantly increased starch and peNDF intake gets overstretched may not be definite and likely animal specific, but our analysis emphasizes the impact of DMI on high SARA susceptibility.

In the first view, it also seems paradoxical that sorting against fine particles was predicted to increase the odds for high SARA severity. But this can be explained by the fact that first measurements on feed particle sorting behavior took place three weeks after the experiment has started and when HIGH cows were already in obvious SARA. Thus, sorting against fine particles may actually be interpreted as a response towards the low ruminal pH and not a cause. Absolute propionate and relative acetate concentrations in the rumen constituted as well influencing factors with more ruminal propionate and less acetate being associated with a high risk for SARA severity. As all cows were fed the same diets in the experiment, these data indicate that the metabolic activity of the rumen microbiome should have been different between SARA types, representing an important risk factor for SARA, which needs to be elucidated in future microbiome-related research.

A last goal of this study was to investigate the consequences of the different SARA severities on various parameters of animal behavior and health as well as rumen and hindgut fermentation in first-lactation Holstein cows. We indeed found that the SARA-type significantly affected intensity and pattern of ruminal fermentation, especially in total VFA concentration and the proportions of propionate and *n*-valerate at the expense of acetate. Interestingly, we found that those discrepancies between SARA types were already present during the close-up feeding while feeding a rather low starch level (i.e., 18.1% in DM). The distinct proportions of acetate and propionate provide evidence for an altered ruminal microbiome between HIGH and LOW cows. Propionate is produced either from succinate or by acrylyl-CoA pathway. The latter is common during high-grain feeding, when propionate is mainly formed from lactate and the acrylate pathway is only expressed by certain bacteria in the rumen, such as *Megasphaera elsdenii* and *Clostridium propionicum*. Although the microbiome analysis should provide better knowledge on this aspect, it is reasonable to assume that the HIGH cows potentially harbored a distinct ruminal microbiome primed on starch utilization and able to harvest more energy from the same diet. Indeed, variations in the ruminal microbiome are also contributory for varying methane emissions of ruminants (Tapio et al., 2017). Hence, a relation of the ruminal microbiome with SARA severity appears plausible and may explain why ruminal propionate and acetate were both identified as influencing factors in our risk analysis. Similarly, Yang et al. (2022) observed more in vitro VFA production with ruminal fluid from susceptible than from resistant cows, which is matching the higher ruminal fermentative intensity in HIGH cows.

Worth of remark is the overall similarity in fecal fermentation pattern between the SARA-types. An increased escape of fermentable substrate from the rumen is typical for grain-induced SARA conditions (Plaizier et al., 2018), especially with high DMI. Thus, one may assume that more fermentable substrate, including starch, entered the hindgut of cows that experience SARA with higher duration and severity. This, in turn, would mean a more extensive hindgut fermentation in HIGH cows than in other cows, potentially resulting in hindgut acidosis. However, the lack of differences in fecal VFA profile and pH indicated an equal amount and composition of post-ileal digesta for all cows. Presumably because HIGH cows had a stronger starch fermentation in the rumen, this levelled out the post-ruminal flow. Likewise, we did not measure fecal pH values < 6.4, leading to the conclusion that the cows did not suffered from hindgut acidosis at any time during the experiment (Enemark, 2008).

Another finding was that the SARA type had marginal impact on the behavior of the cows. Indeed, we found no substantial differences in feed particle sorting behavior between HIGH and LOW cows, only that HIGH cows slightly tended to sort more against fine particles than LOW cows. This indicated a compensation mechanism for relieving the acidotic insult and has been described before in SARA-challenged primiparous cows (Stauder et al., 2020). However, the extent of sorting in our study was not enough to explain the observed differences in SARA severity between the SARA-types. Likewise, chewing behavior served only partially as an explanation for the variance in SARA severity. When related to DMI, the HIGH cows ruminated less after parturition compared to the close-up period, suggesting a lower secretion of rumen-buffering saliva (Zebeli et al., 2015). However, the values of the HIGH cows were not distinct from those of the more resistant SARA-types and the total rumination time did further not undercut the critical threshold of 8 h/d (Paudyal, 2021). In fact, total rumination time was similar in all SARA-types with moderate- or high-grain feeding in 3 wk ap and 7 wk pp, respectively. Consequently, our data do not support the hypothesis of less rumination activity in the HIGH SARA-type, nor do they confirm prior research suggesting that SARA severity was characterized by reduced rumination activity (Khiaosa-Ard et al., 2018). Still, chewing activity was measured 7 wk after switching from moderate- to high-grain feeding. It is indeed conceivable that differences were stronger directly after diet change and then diminished (Kröger et al., 2017).

In evaluating the impact of SARA severity on systemic inflammation, we detected that SARA-type did not affect blood concentrations of systemic inflammation markers. Taking into account that HIGH cows experienced extended and severe SARA, this observation was surprising and contradicted our hypothesis. Periods of SARA typically lead to impaired epithelial barrier function and in consequence pronounced translocation of endotoxins into the bloodstream, eventually increasing APP concentrations (Plaizier et al., 2018; Aschenbach et al., 2019). In the present study, neither haptoglobin nor SAA were different between SARA-types and the systemic health of all cows appeared not detrimentally impacted. Therefore, also HIGH cows seemed to be able to compensate the commonly assumed impairments of prolonged SARA events on systemic inflammation. This observation, however, has been described before in literature when SARA was induced either by feeding starch-rich diets supplemented with pelleted alfalfa (Khafipour et al., 2009), or by feeding starch-rich diets with prolonged feeding challenges (Qumar et al., 2017). The increased APP concentration early pp may be interpreted as a physiological protective reaction of the cows’ innate immune system against immunogenic substances exposed in the events accompanying parturition (Regassa and Noakes, 1999; Sheldon et al., 2008). Still, the stronger magnitude of ruminal pH drop pp, observed in the present study and in others (Penner et al., 2007), may also explain the elevated APP in that time. On the other hand, it is also possible that an activated innate immune system of the cows around parturition might have led to development of a state of immune tolerance due to the sustained inflammation (Galletti et al., 2017), so that no innate immune response was observed when cows were then exposed to high-grain diets and during excessive ruminal pH depression. Nevertheless, further research is warranted to elucidate the immune responses of SARA in particular in postpartum cows.

The lacking influence of SARA-type on lying behavior seems explainable by the slow and insidious pathogenesis of feeding-induced laminitis that leads to extended lying durations of dairy cows (Nocek, 1997). Indeed, changes in lying behavior on a short-term perspective are uncommon (DeVries et al., 2009), but we recently reported poor claw health in primiparous dairy cows around 3 mo after the high-grain feeding period (Kofler et al., 2023). Thereby, high SARA severity was associated with higher incidences of lameness and white line lesions. Consequently, despite the absence of inflammatory symptoms in all cows, a subclinical translocation of endotoxins into the systemic circulation might have taken place in HIGH cows during and after the present study.

The course of liver enzyme concentrations indicated compromised liver health with ongoing high-grain feeding for all cows. A stronger hepatic burden has been associated with higher SARA severity in high-grain-fed primiparous cows (Stauder et al., 2020), which seems not true for our study. Instead, a generally depleted compensation capacity of the liver seems more likely. It is known that the high fat mobilization in early lactation as well affect liver health due to excessive lipid deposition and lesions in the hepatic tissue (Humer et al., 2015), which additionally could have contributed to the increments of liver enzymes observed soon after calving.

## Conclusions

Our study revealed different SARA severity in first-lactation Holstein cows transitioned from a close-up diet to a high-grain diet during early lactation. Apart from nutritional factors, our results highlight the strong relevance of herd management factors for SARA prevention. Moreover, SARA severity was further influenced by high DMI as well as ruminal propionate and acetate, suggesting that the ruminal microbiome’s metabolic function constitutes an influencing factor of SARA, too. The longer and more severe SARA conditions in HIGH primiparous cows were not related to animal behavior and all cows showed physiological rumination activity. Similarly, the distinct SARA-types were not reflected in differences in inflammation and liver health variables. Also, there were no indications of hindgut acidosis, while liver health seemed generally compromised with an ongoing duration of high-grain feeding during lactation. We acknowledge that the study size is limited, but represents a comprehensive long-term study covering the period from transition until end of early lactation. Larger studies with bigger animal cohorts will be needed to validate our findings, especially the risk factors for high SARA severity.

## Supplementary Material

skae041_suppl_Supplementary_Figure_S1-S5

skae041_suppl_Supplementary_Table_S1

## Abbreviations

δ: minimal detectable difference
AUC: area under the curve
ADFom: acid detergent fiber expressed exclusive of residual ash
aNDFom: neutral detergent fiber assayed with a heat stable α-amylase and expressed exclusive of residual ash
ap: antepartum
APP: acute-phase protein
AST: aspartate aminotransferase
GGT: γ-glutamyl-transferase
GLDH: glutamate dehydrogenase
DM: dry matter
DMI: dry matter intake
OR: odds ratio
peNDF>_8_: physically effective NDF larger than 8 mm
pp: postpartum
SAA: serum amyloid A
SARA: subacute rumen acidosis
TMR: total mixed ration
VFA: volatile fatty acids
