# Supplementary material for: Elucidating the factors and consequences of the severity of rumen acidosis in first-lactation Holstein cows during transition and early lactation
Source: J Anim Sci. 2024 Feb 14;102:skae041. doi: 10.1093/jas/skae041 (PMC10946224; doi:10.1093/jas/skae041)
Supplement: skae041_suppl_Supplementary_Figure_S1-S5 [file skae041_suppl_supplementary_figure_s1-s5.docx]

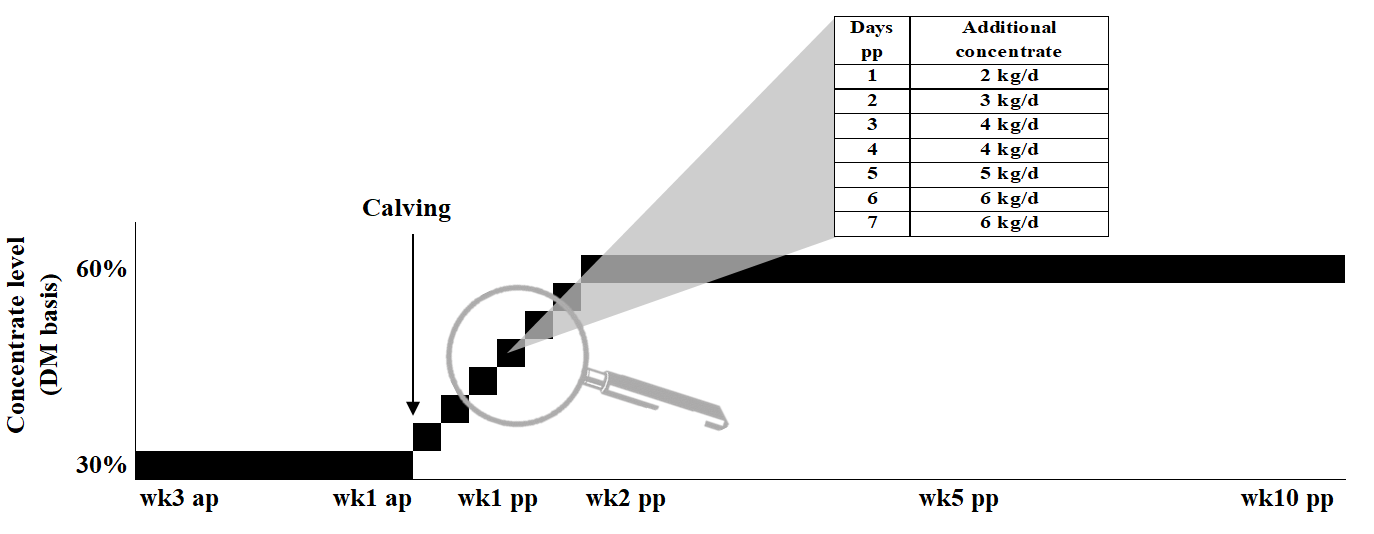


**Supplementary Figure 1.** Overview of gradual increments in concentrate in the diet fed to cows over the first week post-calving.

**
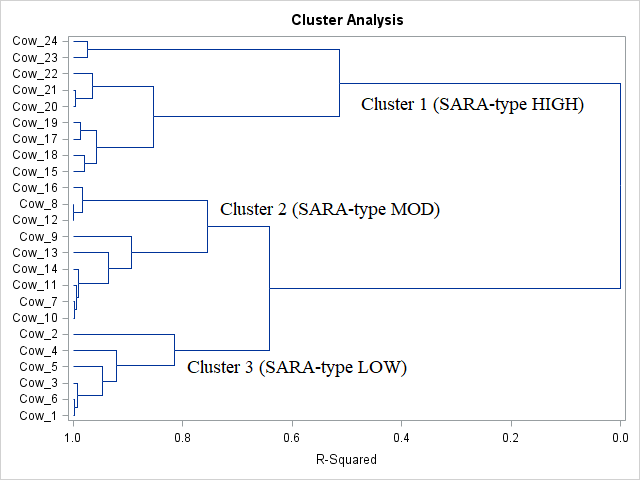
**

**Supplementary Figure 2.** Dendrogram of the cluster analysis revealing 3 SARA-types among cows.


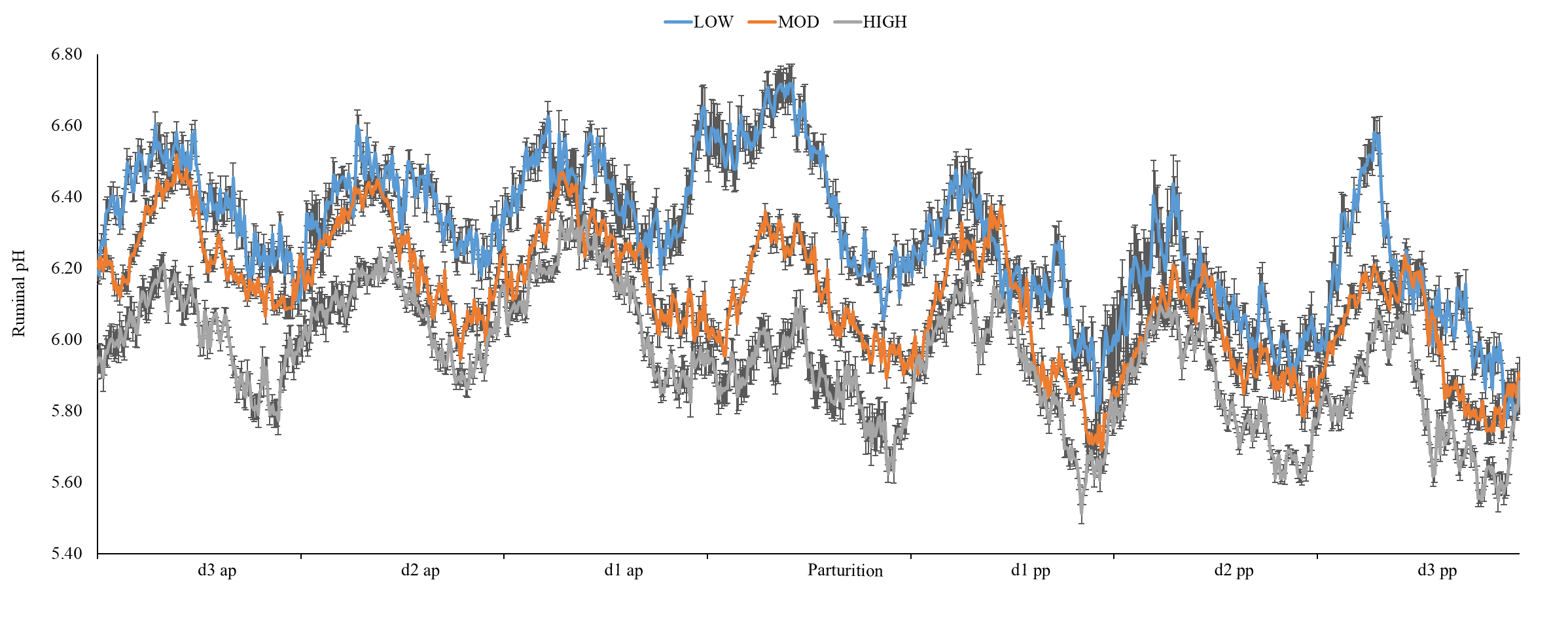


**Supplementary Figure 3.** Ruminal pH in 10 min interval resolution of first-lactation Holstein cows with low (LOW, blue), moderate (MOD; orange), and high (HIGH, grey) SARA severity from 3 days before (ap) until 3 days after parturition (pp). Lines illustrate means with respective standard errors.

**
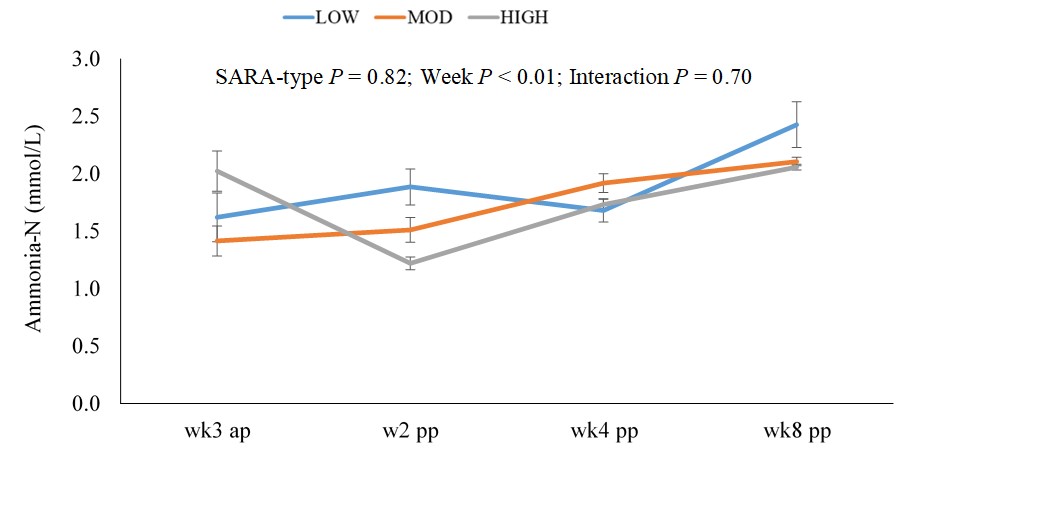
**

**Supplementary Figure 4.** Ruminal ammonia-N profiles of first-lactation Holstein cows with low (LOW, blue), moderate (MOD; orange), and high (HIGH, grey) SARA severity at different weeks before (ap) and after parturition (pp). Lines illustrate least square means with respective standard errors.

**
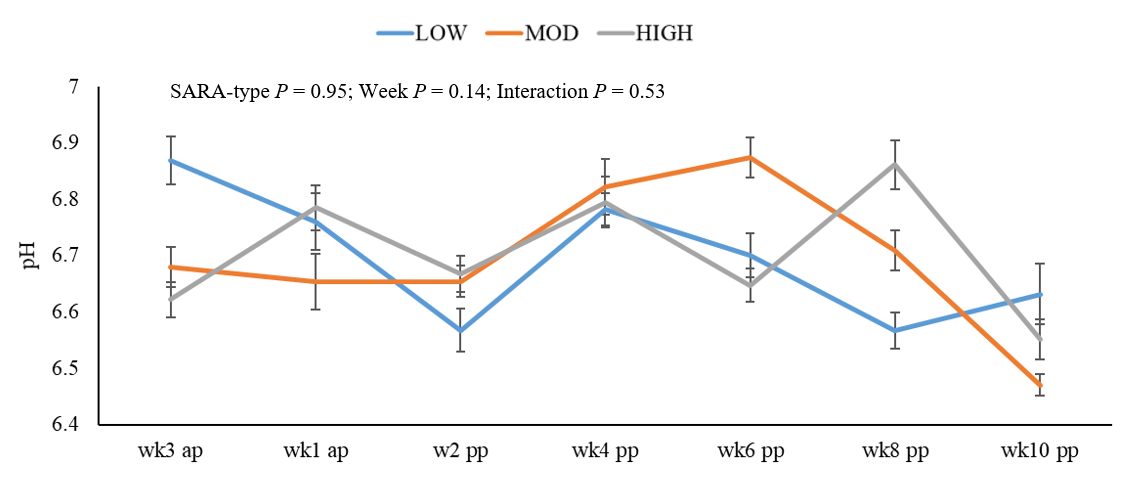
**

**Supplementary Figure 5.** Fecal pH profiles of first-lactation Holstein cows with low (LOW, blue), moderate (MOD; orange), and high (HIGH, grey) SARA severity in different weeks before (ap) and after parturition (pp). Lines illustrate least square means with respective standard errors.
