# Supplementary material for: Elucidating the factors and consequences of the severity of rumen acidosis in first-lactation Holstein cows during transition and early lactation
Source: J Anim Sci. 2024 Feb 14;102:skae041. doi: 10.1093/jas/skae041 (PMC10946224; doi:10.1093/jas/skae041)
Supplement: skae041_suppl_Supplementary_Table_S1 [file skae041_suppl_supplementary_table_s1.docx]

**Supplementary Table 1.** Fecal volatile fatty acid (VFA) profile in first-lactation Holstein cows relative to calving time and differing in SARA severity^1^.

|  | Wk3 ap^2^ | | | Wk1 ap | | | Wk2 pp^3^ | | | Wk4 pp | | | Wk6 pp | | | Wk8 pp | | | Wk10 pp | | |  | *P*-values | | |
| --- | --- | --- | --- | --- | --- | --- | --- | --- | --- | --- | --- | --- | --- | --- | --- | --- | --- | --- | --- | --- | --- | --- | --- | --- | --- |
|  | LOW | MOD | HIGH | LOW | MOD | HIGH | LOW | MOD | HIGH | LOW | MOD | HIGH | LOW | MOD | HIGH | LOW | MOD | HIGH | LOW | MOD | HIGH | SEM | SARA-Type | Week | Interaction |
| Total VFA, µmol/mL | 35.5 | 46.8 | 54.0 | 49.9 | 52.2 | 50.1 | 77.7 | 79.1 | 72.6 | 78.1 | 87.3 | 86.1 | 68.7 | 86.7 | 94.6 | 72.4 | 77.0 | 71.9 | 65.6 | 78.2 | 77.6 | 7.16 | 0.12 | <0.01 | 0.42 |
| Acetate, % | 73.8^bB^ | 76.5^a^ | 75.8^ab^ | 77.7^A^ | 77.2 | 76.0 | 74.5^AB^ | 74.7 | 74.7 | 76.6^A^ | 74.6 | 75.8 | 75.8^AB^ | 75.0 | 75.7 | 75.4^AB^ | 75.0 | 75.5 | 73.3^B^ | 74.5 | 74.9 | 0.63 | 0.82 | <0.01 | <0.01 |
| Propionate, % | 16.9 | 16.1 | 16.5 | 14.5 | 15.3 | 16.1 | 15.2 | 16.1 | 15.4 | 13.8 | 15.3 | 14.5 | 14.5 | 14.8 | 14.3 | 14.8 | 14.8 | 14.9 | 15.6 | 15.3 | 15.0 | 0.62 | 0.56 | <0.01 | 0.57 |
| n-Butyrate, % | 5.05 | 4.81 | 4.65 | 5.00 | 4.66 | 4.95 | 6.56 | 5.33 | 5.73 | 5.61 | 5.43 | 5.69 | 5.83 | 5.90 | 6.56 | 6.61 | 6.53 | 6.06 | 6.71 | 6.19 | 6.31 | 0.36 | 0.28 | <0.01 | 0.25 |
| n-Valerate, % | 1.45^A^ | 1.10 | 1.17 | 1.19^AB^ | 1.14 | 1.24 | 1.02^AB^ | 1.16 | 1.18 | 0.97^B^ | 1.22 | 1.04 | 1.05^AB^ | 1.17 | 1.08 | 0.99^B^ | 1.21 | 1.16 | 0.90^B^ | 1.16 | 1.15 | 0.10 | 0.35 | 0.10 | 0.02 |
| iso-Butyrate, % | 1.74 | 0.98 | 1.20 | 0.93 | 1.08 | 1.00 | 2.15 | 2.00 | 2.23 | 2.43 | 2.74 | 2.38 | 2.13 | 2.56 | 2.01 | 1.80 | 2.01 | 1.84 | 2.73 | 2.32 | 2.18 | 0.31 | 0.52 | <0.01 | 0.58 |
| iso-Valerate, % | 1.05^aA^ | 0.45^b^ | 0.65^ab^ | 0.58^AB^ | 0.38 | 0.58 | 0.45^B^ | 0.66 | 0.64 | 0.52^AB^ | 0.63 | 0.49 | 0.56^AB^ | 0.52 | 0.28 | 0.33^B^ | 0.39 | 0.43 | 0.66^AB^ | 0.44 | 0.32 | 0.12 | 0.15 | <0.01 | <0.01 |

^1^SARA severity based on a cluster analysis considering rumen pH metrics and resulted in 3 distinct severities, i.e., low (LOW), moderate (MOD), and high (HIGH) SARA severity; ^2^Antepartum; ^3^Postpartum.

Capitalized superscript letters indicate differences between weeks within the same SARA-type (*P <* 0.05).

Lower case superscript letters indicate differences between SARA-Type within the same week. (*P <* 0.05).
